# Cardiac arrest patients admitted to intensive care unit after cardiopulmonary resuscitation: a retrospective cohort study to find predictors for mortality

**DOI:** 10.1016/j.bjane.2021.03.013

**Published:** 2021-04-20

**Authors:** Kaan Katircioglu, Pinar Ayvat, Fatma Gunturkun

**Affiliations:** a Izmir Tinaztepe University, School of Medicine, Izmir, Turkey; b Izmir Democracy University, Izmir, Turkey; c University of Tennessee, Health Science Center, College of Medicine, Knoxville, United States

**Keywords:** Critical care, Cardiac arrest, Resuscitation, Prognosis

## Abstract

**Background:**

In-hospital cardiac arrest is a common situation in hospital settings. Therefore, healthcare providers should understand the reasons that could affect the results of cardiopulmonary resuscitation. We aimed to determine the independent predictors for poor outcomes after the return of spontaneous circulation in in-hospital cardiac arrest patients, and also look for a relationship between patient...s background parameters and the status at intensive care unit.

**Methods:**

We did a retrospective cohort study using cardiac arrest patients admitted to the intensive care unit after successful cardiopulmonary resuscitation between 2011...2015. Patients... data were gathered from hospital database. Estimated probabilities of survival were computed using the Kaplan-Meier method. Cox proportional hazard models were used to determine associated risk factors for mortality.

**Results:**

In total, 197 cardiac arrest patients were admitted to anesthesia intensive care unit after successful cardiopulmonary resuscitation in a 4-years period. Of 197 patients, 170 (86.3%) died in intensive care unit. Median of survival days was 4 days. Comorbidity (*p*.ß=.ß0.01), higher duration of cardiopulmonary resuscitation (*p*.ß=.ß0.02), lower Glasgow Coma Score (*p*.ß=.ß0.00), abnormal lactate level (*p*.ß=.ß0.00), and abnormal mean blood pressure (*p*.ß=.ß0.01) were the main predictors for increased mortality in cardiac arrest patients after intensive care unit admission.

**Conclusion:**

The consequent clinical status of the patients is affected by the physiological state after return of spontaneous circulation. Comorbidity, higher duration of cardiopulmonary resuscitation, lower arrival Glasgow Coma Score, abnormal lactate level, and abnormal mean blood pressure were the main predictors for increased mortality in patients admitted to the intensive care unit after successful cardiopulmonary resuscitation.

## Introduction

In-hospital cardiac arrest (IHCA) is a common situation in hospital settings. After a sudden cardiac arrest (CA), mortality rate is considerably high.^1^, ^2^ About 200,000 patients per year were hospitalized for treatment of CA in the USA; and the survival to hospital discharge rate was reported as 7...26%.^3^, ^4^ Although there have been improvements in healthcare, patient situation after CA does not seem to improve considerably. There are various factors associated with poor patient outcome after CA. Therefore, healthcare providers should understand the reasons that could affect the results of cardiopulmonary resuscitation (CPR).

We aimed to determine the independent predictors for poor outcomes after return of spontaneous circulation (ROSC) in IHCA patients, and to look for a relationship between patient...s background parameters and the status at intensive care unit (ICU).

## Methods

The University of Izmir Katip Celebi Institutional Review Board approved all aspects of this study (20.12.2017 Protocol number: 316). Since this is a retrospective cohort study, Institutional Review Board did not require the written informed consent.

Izmir Ataturk Training and Research Hospital (IATRH) is a research and training hospital in Izmir Turkey with over 50,000 hospital admissions per year and a 1400-bed tertiary care referral center affiliated with Izmir Katip Celebi University. The department of anesthesiology has an ICU with a total number of 31 beds. Our ICU is a level 3 ICU and not only provides perioperative care but also primary ICU care for all kinds of adult patients.

**Figure 1:**
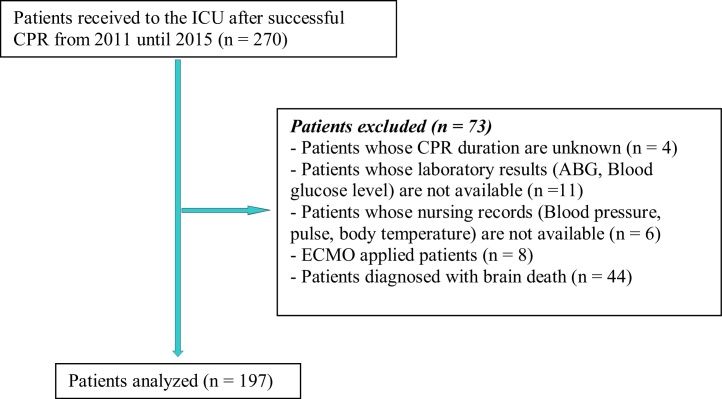
Flow diagram of the study.

We did this retrospective cohort study using CA patients received to the ICU of IATRH after successful CPR from 2011 until 2015. Patient data were gathered from hospital database and ICU patient records. Our inclusion criteria were successful resuscitation from CA after ROSC. We did not include patients of whom we do not know the time of arrest, as well as arterial blood gas (ABG) and physiologic values were unavailable after ROSC. We also excluded the patients for whom extracorporeal membrane oxygenation (ECMO) was used. In addition, patients diagnosed with brain death were not included in this study. Flow diagram of the study is presented in Fig. 1. We used survival to hospital discharge as the primary outcome measure. Because this is a retrospective cohort study from 2011 until 2015, sample size was not calculated.

Demographic characteristics of the patients, admission sources, arrival Glasgow Coma Score (GCS), comorbidity, duration of CPR, ABG, mean blood pressure (MBP), pulse rate (PR), serum glucose levels, patient temperatures, survival periods (days) were analyzed.

Analyzed parameters were defined as: demographic characteristics (age, sex); admission source (operating room or other (emergency department, ward)); comorbidity (cardiac, respiratory, neurologic, renal, hepatic, malignancy, diabetic, intoxication) (no comorbidity, 1...2 comorbidities, or 3 or more comorbidities); duration of CPR (below 20 minutes or above); GCS (3 or above); ABG ... pH (between 7.35...7.45 or other), PaO_2_ (between 80...100.ßmmHg or other), PaCO_2_ (between 35...45.ßmmHg or other), lactate levels (below 4.ßmmol.L^-1^ or above); hemodynamic parameters ... MBP (between 80...119.ßmmHg or other), PR (between 60...100/min or other); temperature (between 36.5...37.ß..C or other); serum glucose levels (between 60...110.ßmmol.L^-1^ or other).

The first measurement for each physiologic value on ICU arrival were included in the analysis. We calculated frequencies and percentages for categorical variables as well as median and range for continuous variables. Estimated probabilities of survival were computed using the Kaplan Meier method. We used log rank test to evaluate the differences between survival distributions in univariate analysis. We made use of cox proportional hazard models to find out associated risk factors for mortality. For all analysis, we used the statistical software RStudio (rstudio.com, open-source software). We considered the results of statistical tests to be significant if two-sided *p*-value is < 0.05.

## Results

One thousand five hundred sixty-two patients were accepted to the ICU during 4 years, starting 2011 through 2015. During this period, a total of 197 CA patients who met our inclusion criteria were analyzed. Baseline characteristics, blood gas analysis, vital signs, and physiologic values of patients are presented in Table 1. Out of these 197 patients, 27 (13.7%) patients survived and were discharged from the critical care unit whereas 170 (86.3%) died in ICU. Of these 170 patients who died, 115 (58.4%) patients, in fact, survived the first day of hospitalization. Eighty-one (41.1%) patients survived the first 7 days of hospitalization. Median of survival days was 4 days (95% CI: 2...6). More than half of all patients (51.3%) were male. Survival rate within the same day was 60.4% in males and 56.3% in females. The median age of the patients was 70 (Range: 21...93). The median ages of survivors and non-survivors were 68 and 71 years, respectively. The admission rate from the operating room was 8.1%. The rate of the patients had no comorbidity was 10.2% whereas 89.8% had multiple comorbidities. In this context, 71.5% had 1...2 and 18.3% had 3 and above multiple comorbidities. While 59 (29.9%) patients were resuscitated for 20 minutes or less, the others were resuscitated for more than 20 minutes. The median of survival days was 7 days (95% CI: 3...9) in patients resuscitated for 20 minutes or less, and 1 day (95% CI: 1...3) in patients resuscitated for more than 20 minutes. Majority of the patients (70.1%) had a GCS of 3 at the admission in ICU. Survival rate of the patients who had a GCS of 3 was 3.6% and 37.3% in other patients. Median lactate level was considerably higher in non-survivors. We calculated median survival time as 14 days (95% CI: 8...17) in patients with normal lactate levels and 1 day (95% CI: 1...2) with abnormal lactate levels. Survival rate within the same day was 91.5% at normal MBP levels and 52.6% at abnormal MBP levels.

**Table 1:** Baseline characteristics, blood gas analysis, vital signs, and physiologic values of patients.

Variable	n (%)	Non-survivors (%)	Survivors (%)	Survivors after the first day (%)	Survivors after 7 days (%)	Median survival time ... days (95% CI)	*p*-value univariate
**Total**	**197**	**170 (86.3)**	**27 (13.7)**	**115 (58.4)**	**81 (41.1)**	**4 (2...6)**	
***Sex***							**0.76**
Male	101 (51.3)	88 (87.1)	13 (12.9)	61 (60.4)	42 (41.6)	4 (2...8)	
Female	96 (48.7)	82 (85.4)	14 (14.6)	54 (56.3)	39 (40.6)	3 (1...7)	

***Age (year)* ... *Median (range)***	**70 (21...93)**	**71 (22...93)**	**68 (21...83)**				**0.3**
< 65 year	62 (31.5)	51 (82.3)	11 (17.7)	39 (62.9)	25 (40.3)	4 (1...8)	
65 and above	135 (68.5)	119 (88.2)	16 (11.8)	76 (56.3)	56 (41.5)	3 (1...7)	

***Admission source***							**0.19**
Operating Room	16 (8.1)	11 (68.8)	5 (31.2)	11 (68.8)	9 (56.3)	10 (1...NA)	
Other	181 (91.9)	159 (87.9)	22 (12.1)	104 (57.5)	72 (39.8)	3 (1...5)	

***Comorbidity***							**0.01**^a^
No	20 (10.2)	14 (70)	6 (30)	12 (60)	11 (55.0)	8 (0...134)	
1...2	141 (71.5)	121 (85.8)	20 (14.2)	87 (61.7)	60 (42.6)	4 (2...7)	
3 and above	36 (18.3)	35 (97.2)	1 (2.8)	16 (44.4)	10 (27.8)	1 (1...4)	

***Duration of CPR (minute)***							**0.02**^a^
Other	59 (29.9)	56 (94.9)	3 (5.1)	24 (40.7)	16 (27.1)	1 (1...3)	
20 minutes and below	135 (68.5)	111 (82.2)	24 (17.8)	89 (65.9)	65 (48.1)	7 (3...9)	
Missing	3 (1.5)						

***GCS***							**0.00**^a^
3	138 (70.1)	133 (96.4)	5 (3.6)	73 (52.9)	43 (31.2)	2 (1...4)	
Other	59 (29.9)	37 (62.7)	22 (37.3)	42 (71.2)	38 (64.4)	9 (7...34)	

***Arterial Ph ... Median (Range)***	**7.23 (6.8...7.6)**	**7.2 (6.8...7.6)**	**7.33 (7...7.6)**				**0.42**
7,35...7,45	37 (18.8)	28 (75.7)	9 (24.3)	31 (83.8)	20 (54.1)	8 (4...14)	
Other	141 (71.6)	123 (87.2)	18 (12.8)	79 (56)	56 (39.7)	3 (1...6)	
Missing	19 (9.6)						

***Lactate (mmol.L-1)* ... *Median (range)***	**4.6 (0.4...27)**	**5.7 (0.4...27)**	**2 (0.5...10.3)**				**0.00**^a^
< 4.ßmmol.L^-1^	73 (37.1)	55 (75.3)	18 (24.7)	64 (87.5)	49 (67.1)	14 (8...17)	
Other	85 (43.1)	78 (91.8)	7 (8.2)	36 (42.4)	23 (27.1)	1 (1...2)	
Missing	39 (19.8)						

***PR* ... *Median (range)***	**100 (45...171)**	**100 (45...171)**	**94 (53...153)**				**0.07**
60...100/minute	97 (49.2)	79 (81.4)	18 (18.6)	65 (67.0)	47 (48.5)	7 (3...9)	
Other	86 (43.7)	77 (89.5)	9 (10.5)	49 (57)	33 (38.4)	3 (1...7)	
Missing	14 (7.1)						

***MBP (mmHg) ... Median (range)***							**0.01**^a^
80...119.ßmmHg	47 (23.9)	38 (80.9)	9 (19.1)	43 (91.5)	32 (68.1)	12 (7...20)	
Other	135 (68.5)	117 (86.7)	18 (13.3)	71 (52.6)	48 (35.6)	2 (1...4)	
Missing	15 (7.6)						

***Temperature (0C)* ... *Median (range)***	**34.9 (34...40)**	**35.5 (34...40)**	**36 (35...37.3)**				**0.28**
36.5...*37* ..*C*	15 (7.6)	13 (86.7)	2 (13.3	15 (100)	10 (66.7)	15 (6...17)	
Other	166 (84.3)	141 (84.9)	25 (15.1)	99 (59.6)	70 (42.2)	4 (2...7)	
Missing	16 (8.1)		0				

***Glucose (mmol.L-1)* ... *Median (range)***	**8.2 (0.3...39.8)**	**8.1 (0.3...39.8)**	**8.3 (4.2...28.5)**				**0.37**
3.3...5.6.ßmmol.L^-1^	54 (27.4)	48 (88.9)	6 (11.1)	31 (57.4)	23 (42.6)	4 (1...9)	
Other	142 (72.1)	121 (85.2)	21 (14.8)	84 (59.2)	58 (40.8)	4 (2...7)	
Missing	1 (0.5)						

***PaO_2_ (mmHg)* ... *Median (range)***	**92.5 (16...497)**	**92 (16...497)**	**99 (49...202)**				**0.76**
80...100.ßmmHg	26 (13.2)	22 (84.6)	4 (15.4)	19 (73.1)	14 (53.8)	8 (2...12)	
Other	150 (76.1)	127 (84.7)	23 (15.3)	90 (60.0)	62 (41.3)	4 (2...7)	
Missing	21 (10.7)						

**PCO_2_*(mmHg)* ...*Median (range)***	**37 (17...105)**	**37.5 (18...105)**	**36 (17...95)**				**0.72**
35...45.ßmmHg	50 (25.4)	43 (86)	7 (14)	35 (70)	20 (40.0)	5 (3...8)	
Other	127 (64.5)	107 (84.3)	20 (15.7)	74 (58.3)	55 (43.2)	3 (1...8)	
Missing	20 (10.2)						

a 95% confidence level.

We found comorbidity (*p*.ß=.ß0.01), higher duration of CPR (*p*.ß=.ß0.02), lower GCS (*p*.ß=.ß0.00), abnormal lactate level (*p*.ß=.ß0.00), and abnormal MBP (*p*.ß=.ß0.01) were the main predictors for increased mortality in CA patients after ICU admission. Demographic factors such as age and sex, physiologic values such as temperature, admission source and laboratory results such as glucose, arterial pH, PaCO_2,_ PaO_2_, abnormal PR did not influence mortality significantly.

**Figure 2:**
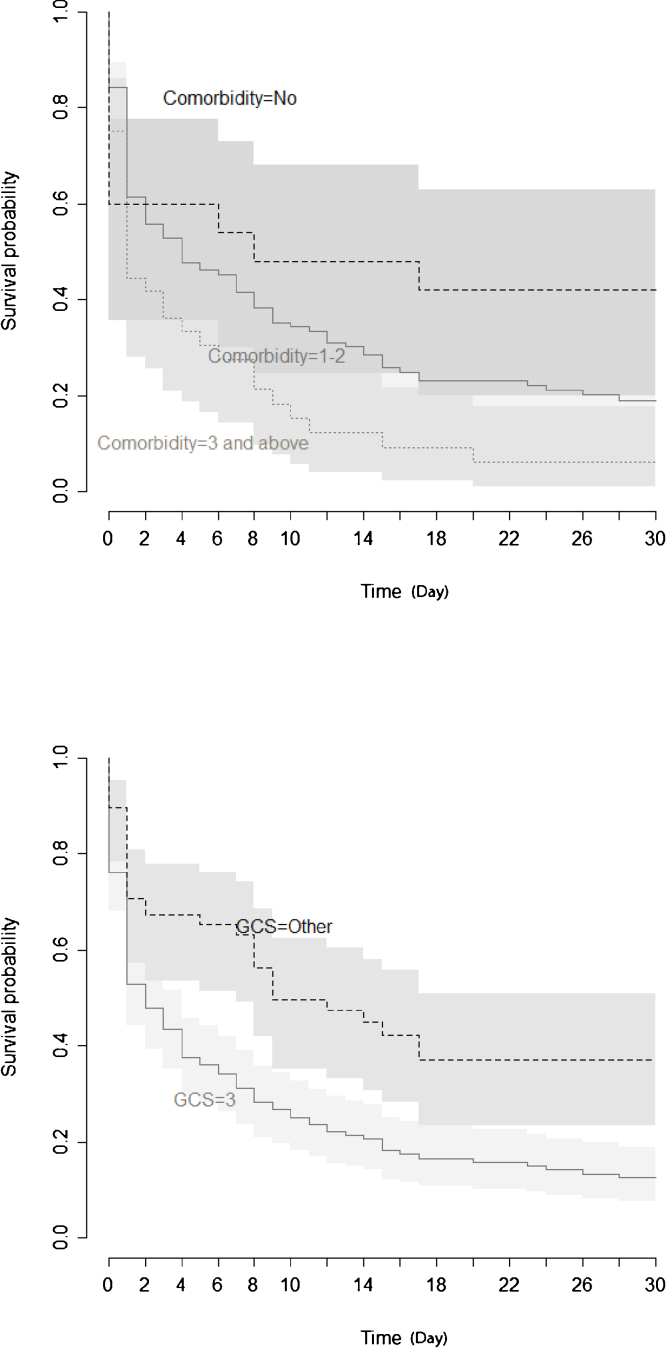
Kaplan Meier estimate of survival after resuscitation from cardiac arrest according to comorbidity and glasgow coma score.

**Figure 3:**
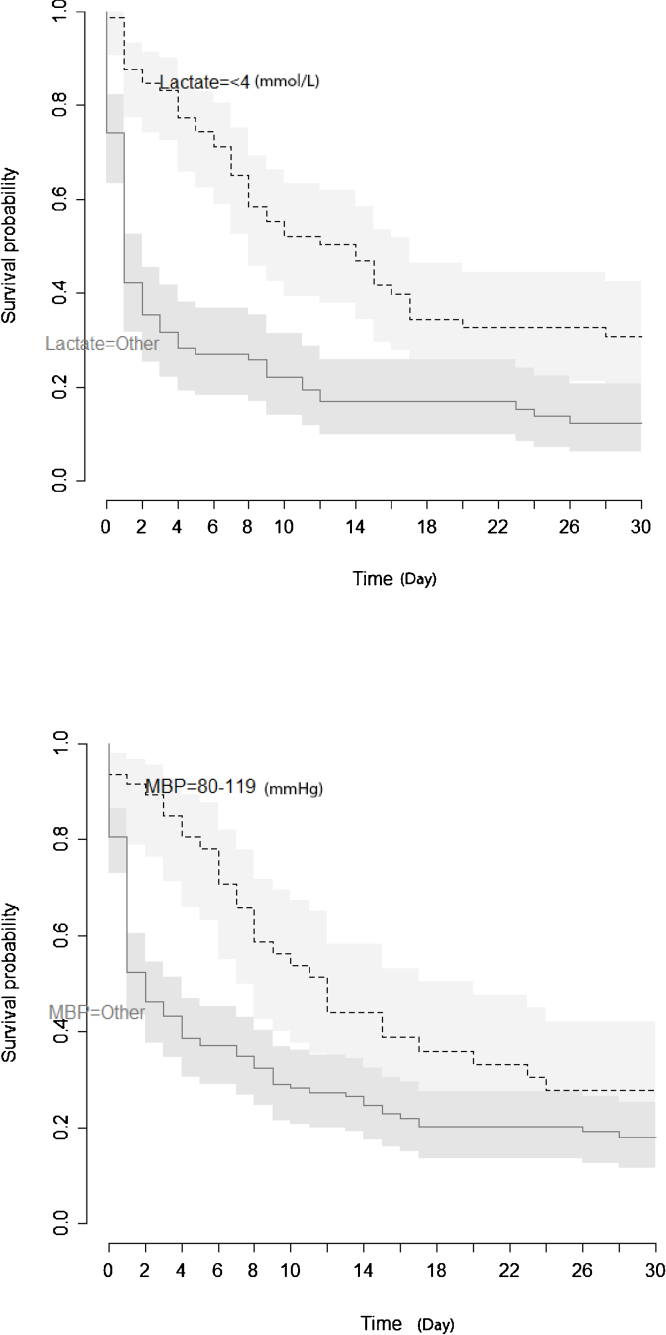
Kaplan Meier estimate of survival after resuscitation from cardiac arrest according to lactate level and mean blood pressure level.

**Figure 4:**
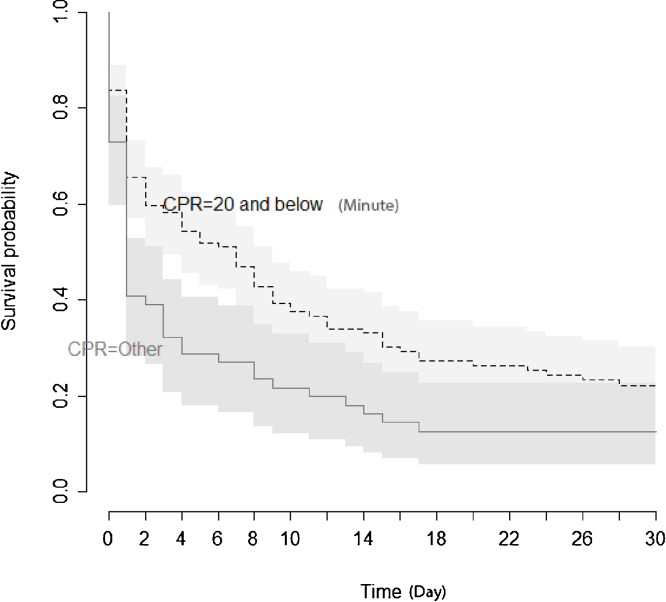
Kaplan Meier estimate of survival after resuscitation from cardiac arrest according to duration of CPR.

Kaplan Meier estimate of survival after CA regarding these predictors for CA mortality are shown in Figure 2, Figure 3, Figure 4. Survival was highest in patients without comorbidity. Mortality risk was beginning to decrease in patients who had a GCS of higher than 3 and lower duration of CPR. There was a notable difference in survival distributions at normal and abnormal levels of lactate and MBP.

We used proportional hazards model to evaluate simultaneous effect of risk factors on survival rates. All variables which were significant in univariate analysis were included in the Cox regression model by backward elimination of non-significant covariates (*p*.ß>.ß0.10). The length of CPR that was significant in univariate analysis was removed from the model. Since the number of patients in the compared groups was very small, the reliability of the cox regression test decreased and could not be calculated. Therefore, we had to perform Cox regression model without duration of CPR. According to the results of the Cox regression analysis, mortality was 1.8 (95% CI: 0.8...3.7) times higher in patients with 1...2 multiple comorbidities and 3.9 (95% CI: 1.7...8.6) times higher in patients with 3 and more multiple comorbidities when compared to those without comorbidity. Lower GCS (HR: 2.7 95% CI: 1.7...4.4) were related with increased mortality. Moreover, the hazard ratios for the abnormal level of lactate and MBP were 2.6 (95% CI: 1.8...3.8) and 2.4 (95% CI, 1.6...3.7), respectively.

GCS at discharge from hospital were 15 in 21 survivors, 10 in 1 survivor, 9 in 1 survivor, 7 in 2 survivors and 6 in 1 survivor.

## Discussion

There are studies that found a relationship between survival after CA and age.^5^, ^6^ Some studies documented a considerably low survival rate for older patients.^7^, ^8^ When these studies are examined, it is seen that there are differences in their methodology and criteria for including patients. Some of them included only adults^5^, ^7^, ^8^ whereas some others included children, too.^6^ We realized that there was no single cut-off value to determine the children from older adults. In our study, age ... 65 was not found to be a negative prognostic factor for the patients who were able to restore blood circulation after CA. The reason behind this may be that the complications leading to CA in younger patients are more serious and harder to reverse compared to older patients.

In most of the previous studies about IHCA, gender does not seem to be associated with survival. Nevertheless, one study^9^ found out that female gender could be a factor for survival after adjustments are done for cardiac rhythm age, reason, and site of arrest. Gender has not been found to be a prognostic factor in our study.

Some research examined the effect of lactate in the post-cardiac arrest population. One of those studies done retrospectively for post-arrest patients found that the outcome of the patient can be predicted by the initial lactate level in the post-arrest period. Mortality was 39% for post-arrest patients that had an initial lactate < 5.ßmmol.L^-1^, on the other hand mortality was as high as 92% for patients who had an initial lactate > 10.ßmmol.L^-1^.^10^ In our study, high lactate levels (> 4.ßmmol.L^-1^) after CA were found to be significantly associated with mortality. Prolonged CA and/or serious consequent hemodynamic failure could be the result of high lactate concentrations.^11^, ^12^, ^13^ Therefore, lactate could be a factor that affects the poor outcomes of the patients. Hence, if there is hemodynamic failure such as CA in patient, it could be better to monitor lactate level rather than just blood pressure or cardiac output. Some research showed that admission blood lactate levels after CA and its fluctuation levels later than CA could be predictive of mortality.^11^, ^12^, ^13^, ^14^

There are a few researches that examined the time length of resuscitation on patient outcome. In their retrospective study, Reynolds et al. showed that with each minute of CPR, the probability of survival to hospital discharge decreased.^15^ After 15 minutes of CPR, the probability of survival decreased to 2% whereas it was 75% for patients who received 10...15 minutes of CPR.^15^ In a similar study, Shih et al. found that patients who received 10 minutes or less of CPR, had a rate of ROSC as high as 90% whereas, ROSC rate for patients who were resuscitated for more than 30 minutes was 50%.^16^ These studies indicate that bad patient outcome is related with the longer duration of resuscitation. In contrast, a study showed that when the time length of resuscitation increased, so did the survival rate for patients who particularly had asystole or preliminary rhythm of pulseless electrical activity.^17^ Lastly, Cha et al. found that if the duration of CPR was more than 30 minutes, the rate of survival was as low as 5.6%.^18^ Obviously, there is no clear cut-off CPR duration in the literature. However, as Rohlin et al. mentioned that a duration longer than 20 minutes captures 95% of the 30-day survivors,^19^ we chose 20.ßminutes as a cutoff time. In our study, a CPR duration over 20 minutes was associated with increased mortality among the patients who achieved ROSC and were admitted to ICU.

The presence of comorbid diseases is related with morbidity. It also affects the functional status and life quality of patients adversely. In their study Fabbri et al. found that pre-arrest comorbidities were associated with decreased chances of survival.^20^ In another study Chakravarthy et al. showed that in CA patients the best survival rate (64%) was among those who had none or 1 comorbidity.^21^ When patients had 2 comorbidities, the chance to survive was 9.6%, whereas no patient survived if they had 2 or more comorbidities.^21^ And finally, Andrew et al. concluded that patients... current comorbidities could help to assess and predict the situations of CA patients.^22^ In our study we found that the survival rate decreased in CA patients among those who had 3 or more comorbidities.

GCS is useful for evaluation of a critical care patient...s status in the face of changing conditions. Recently Martinell et al. identified low ICU arrival GCS as a predictor of a worse patient situation at 6 months for those patients who initially survived out-of-hospital cardiac arrest.^23^ Low arrival GCS (3) was also associated with a poor outcome in our study.

High morbidity and high mortality rates are common after CA. These high rates could be reduced by using mechanical ventilation, maintaining normal hemodynamic parameters, keeping normoxia as well as normocapnia. Recently Sutherasan et al. proposed a general bundle of treatment to enhance the prognosis of patients after CA.^24^ But mostly achieving these normal parameters is difficult in CA patients. In our study, among these parameters abnormal mean arterial blood pressure was the predictor for increased mortality in post-cardiac arrest patients.

This study is limited by the fact that we were not able to provide data of the resuscitation process. Every case of CPR is different from one another, therefore the response in each case could change the outcome of the resuscitation. Additionally, the analysis was retrospective. Our main endpoint was survival to hospital discharge, and did not include data on functional outcome, nor follow-up post discharge.

## Conclusion

The consequent clinical status of the patient is affected by the physiological state after ROSC. Initial examination and laboratory results following ROSC could be useful in providing predictive information for both physicians and patients... families. We found comorbidity, higher duration of CPR, lower arrival GCS, abnormal lactate level and abnormal MBP were the main predictors for increased mortality in patients admitted to the ICU after successful CPR. Demographic factors as age and gender, physiologic value like temperature, laboratory results like blood glucose, arterial pH, PaCO_2,_ PaO_2_, and abnormal PR did not influence mortality significantly.

## Conflict of interest

The authors declare no conflicts of interest.
